# Recent Vibrio cholerae O1 Epidemic Strains Are Unable To Replicate CTXΦ Prophage Genome

**DOI:** 10.1128/mSphere.00337-21

**Published:** 2021-06-09

**Authors:** Kaoru Ochi, Tamaki Mizuno, Prosenjit Samanta, Asish K. Mukhopadhyay, Shin-ichi Miyoshi, Daisuke Imamura

**Affiliations:** a Department of Frontier Bioscience, Hosei University, Koganei, Tokyo, Japan; b Graduate School of Medicine, Dentistry and Pharmaceutical Sciences, Okayama University, Okayama, Japan; c Division of Bacteriology, National Institute of Cholera and Enteric Diseases, Kolkata, India; University of Iowa

**Keywords:** *Vibrio cholerae*, cholera, prophage

## Abstract

Cholera, an acute diarrheal disease, is caused by pathogenic strains of Vibrio cholerae generated by the lysogenization of the filamentous cholera toxin phage CTXΦ. Although CTXΦ phage in the classical biotype are usually integrated solitarily or with a truncated copy, those in El Tor biotypes are generally found in tandem and/or with related genetic elements. Due to this structural difference in the CTXΦ prophage array, the prophage in the classical biotype strains does not yield extrachromosomal CTXΦ DNA and does not produce virions, whereas the El Tor biotype strains can replicate the CTXΦ genome and secrete infectious CTXΦ phage particles. However, information on the CTXΦ prophage array structure of pathogenic V. cholerae is limited. Therefore, we investigated the complete genomic sequences of five clinical V. cholerae isolates obtained in Kolkata (India) during 2007 to 2011. The analysis revealed that recent isolates possessed an altered CTXΦ prophage array of the prototype El Tor strain. These strains were defective in replicating the CTXΦ genome. All recent isolates possessed identical *rstA* and intergenic sequence 1 (Ig-1) sequences and comparable *rstA* expression in the prototype El Tor strain, suggesting that the altered CTXΦ array was responsible for the defective replication of the prophage. Therefore, CTXΦ structures available in the database and literatures can be classified as replicative and nonreplicative. Furthermore, V. cholerae epidemic strains became capable of producing CTXΦ phage particles since the 1970s. However, V. cholerae epidemic strains again lost the capacity for CTXΦ production around the year 2010, suggesting that a significant change in the dissemination pattern of the current cholera pandemic occurred.

**IMPORTANCE** Cholera is an acute diarrheal disease caused by pathogenic strains of V. cholerae generated by lysogenization of the filamentous cholera toxin phage CTXΦ. The analysis revealed that recent isolates possessed altered CTXΦ prophage array of prototype El Tor strain and were defective in replicating the CTXΦ genome. Classification of CTXΦ structures in isolated years suggested that V. cholerae epidemic strains became capable of producing CTXΦ phage particles since the 1970s. However, V. cholerae epidemic strains again lost the capacity for CTXΦ production around the year 2010, suggesting that a critical change had occurred in the dissemination pattern of the current cholera pandemic.

## INTRODUCTION

Cholera is an acute diarrheal disease and remains a major threat to health, particularly in developing countries (1, 2). It is caused by infection with toxigenic Vibrio cholerae strains (3). Although over 200 serogroups of V. cholerae have been identified, 2 serogroups (O1 and O139) are responsible for cholera epidemic and pandemic (4, 5). The serogroup O1 can be further classified into 2 biotypes (classical and El Tor). Toxigenic V. cholerae strains are generated by the infection and lysogenization of a filamentous phage, CTXΦ (6). The genome of CTXΦ prophage contains *ctxAB*, which encodes cholera toxin, which is the primary virulence factor of cholera responsible for severe and watery diarrhea. Since 1817, seven pandemics of cholera have been recorded. The sixth and, presumably, the earlier pandemics emerged from the Gangetic delta and were caused by the classical biotype of V. cholerae, whereas the current ongoing seventh pandemic has been attributed to the El Tor biotype (5). The V. cholerae El Tor biotype has shown remarkable changes over the years of the seventh pandemic. A new pathogenic variant that possesses the classical type *ctxB* (*ctxB1*) with an El Tor type genomic backbone has emerged (7–10). More recently, the novel *ctxB* variant (*ctxB7*) has been found in Haiti and other countries (11, 12). Ghosh et al. demonstrated that the Haitian variant strain has evolved because of sequential events on the Indian subcontinent with some cryptic modification in the genome (13). Moreover, it has also been reported that the seventh pandemic strain first appeared in the Gangetic delta and recurrently spread from this area to the rest of the world, in at least three waves (14). Therefore, the Gangetic delta is considered the epicenter for all cholera pandemics according to the historical records. We recently isolated and characterized V. cholerae strains from patients suffering from cholera who were residing in Kolkata, India, which is a representative area of the Gangetic delta, between 2007 and 2014 (15). The analysis revealed that the cholera epidemics were caused by distinct V. cholerae O1 strains and that the predominant strains have undergone a shift in recent years.

**FIG 1:**
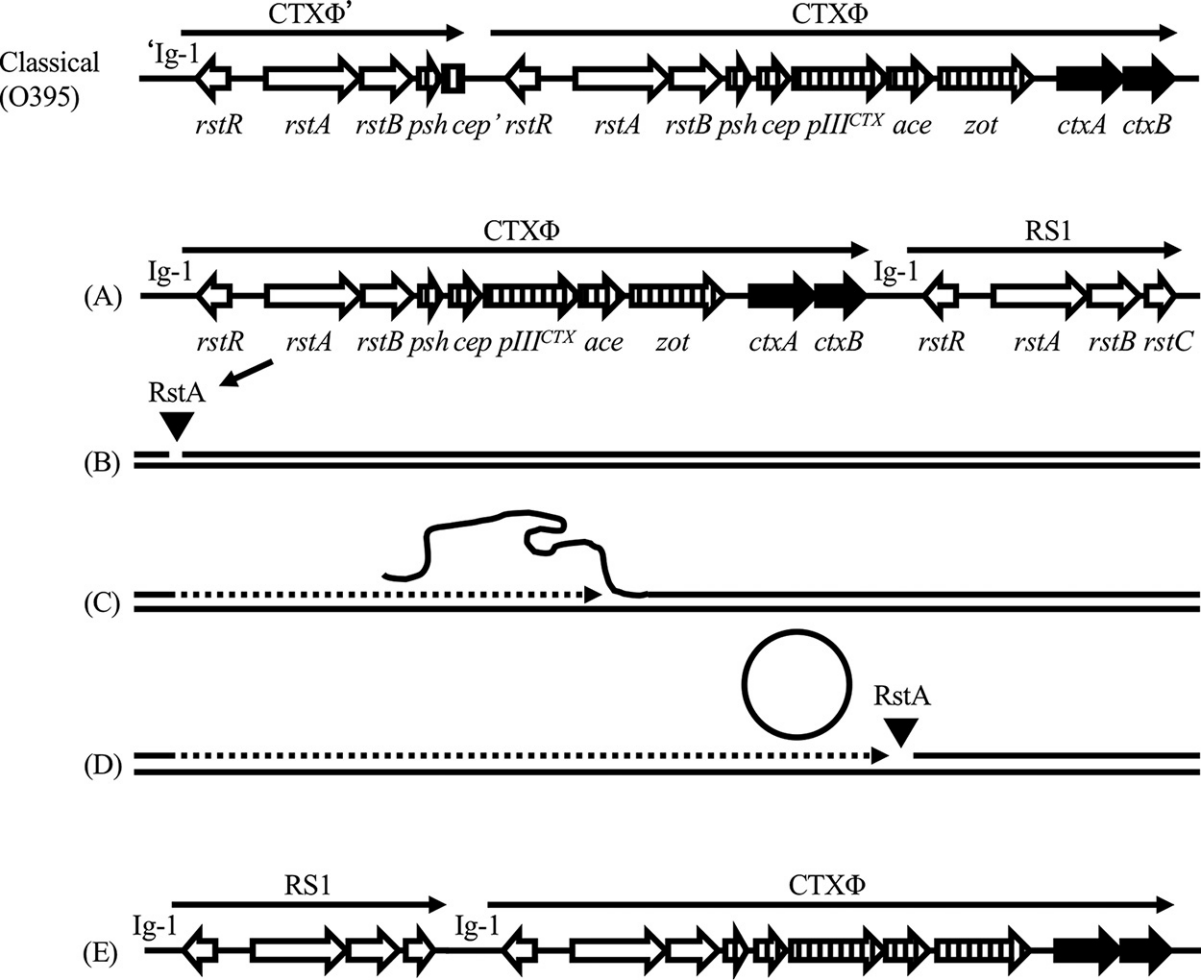
Model for the rolling-circle replication of the CTXΦ prophage genome. Genes in the CTXΦ prophage and its array in the classical biotype O395 and El Tor biotype N16961 are shown (top and panel A). Open arrows indicate the genes necessary for DNA replication and the integration of the phage. Striped arrows indicate the genes required for phage packaging and secretion (21). Solid arrows indicate the genes responsible for encoding the cholera toxin. Ig-1 was located adjacent to *rstR* in CTXΦ and RS1, and the CTXΦ prophage genome was located between the two Ig-1 sequences in the prototype El Tor biotype strain (A). RstA nicked Ig-1 in the plus-strand DNA of CTXΦ (B). Host replication machinery synthesized a new plus strand while displacing the old plus strand (C). RstA nicked Ig-1 at the downstream end of the CTXΦ prophage genome, resulting in a closed circular single-strand DNA (D). RS1 in recent isolates used in this study was located at the upstream end of the CTXΦ genome, compared with the prototype El Tor the biotype strains (E). This type of CTXΦ prophage lacked the Ig-1 at the downstream end of the CTXΦ genome.

Studies have indicated that the biotypes of pathogenic V. cholerae strains have generally been recognized based on the genotypes of pathogenic genes, including *ctxB* and/or phylogeny of the genomic backbone (14, 15). In addition to this analysis, diverse CTXΦ prophage arrays have also been found within strains isolated from epidemics (16). Classical strains usually possess a single CTXΦ genome or a CTXΦ genome with truncated copy on the larger chromosome and also have an additional single CTXΦ genome on the smaller chromosome (17). In two classical biotype strains (O395 and 569B), a truncated CTXΦ comprising *rstR*, *rstA*, and *rstB* and a partial *cep* followed by an intact CTXΦ genome in the larger chromosome (Fig. 1, top) and another CTXΦ prophage in the smaller chromosome are integrated (18). Although CTXΦ prophage genomes of these strains contain intact gene sets, CTXΦ is unable to replicate its genome due to the prophage array structure. The CTXΦ prophage genome in the El Tor biotype strains was usually found in tandem and/or with a related genetic element known as RS1 in the larger chromosome (16, 19). RS1 contains *rstC* and the following genes in CTXΦ phage: *rstR*, *rstA*, and *rstB* (Fig. 1A). CTXΦ prophage DNA is replicated by a rolling-circle mechanism that requires *rstA* (20). RstA nicks the plus strand DNA of the CTXΦ prophage genome at intergenic sequence 1 (Ig-1), which is located adjacent to *rstR* (Fig. 1B). The host replication machinery synthesizes a new plus strand, while displacing the old plus strand (Fig. 1C). Moreover, RstA also nicks the Ig-1 of the adjacent CTXΦ prophage or RS1 downstream of the CTXΦ prophage genome in the El Tor biotype, which releases a closed circular ssDNA (Fig. 1D, Moyer 2001). Thus, Ig-1 both upstream and downstream of the CTXΦ prophage genome (i.e., the presence of tandem elements, the presence of either of the two prophages, or the presence of a prophage followed by an RS1) is necessary for the replication of the CTXΦ genome. As a consequence, CTXΦ phage in the classical biotype strains does not yield extrachromosomal CTXΦ DNA and thus does not produce virions, whereas El Tor biotype strains can secrete infectious CTXΦ particles (17). These observations indicate that the transmission of CTXΦ genome from toxigenic V. cholerae to other strains during the sixth and earlier pandemics was not caused by the phage infection. It was restricted to natural competence or other indirect horizontal transfer of V. cholerae. In contrast, during the seventh pandemic, CTXΦ phage particles were produced from toxigenic V. cholerae and probably generated new toxigenic strains, spreading the infection in the environment. Hence, the dissemination and continuation patterns of cholera pandemics are dissimilar.

Recently we reported that two distinct lineages of pathogenic V. cholerae strains were concurrently prevalent between 2007 and 2009, while one lineage became predominant in 2010 and later in Kolkata, India (15). These investigations were performed by phylogenetic analyses, based on the single nucleotide polymorphisms of core genomic sequences, using short reads obtained with an Illumina next-generation sequencer. However, these analyses do not provide insights with respect to the copy number and array of repeated genomic sequences, which play important roles in the virulence of V. cholerae (21). To investigate the variations in the chromosomal structures of V. cholerae epidemic strains, including copy numbers and the arrangement of repeated sequences, we developed complete genomic sequences of five representative V. cholerae strains isolated from Kolkata, India, in this study. The analysis demonstrated that the recent epidemic strains of V. cholerae El Tor biotype possess both intact CTXΦ prophage and RS1, although in the altered array of prototype El Tor strains. The strains with an altered CTXΦ array were incapable of replicating the CTXΦ genome, suggesting that a critical change may have occurred in the dissemination and continuation route of the current cholera pandemic.

## RESULTS

### Structural variations in the genomes of recent V. cholerae epidemic strains.

It was reported that two distinct lineages (1 and 2) of pathogenic V. cholerae strains were concurrently prevalent between 2007 and 2009, while lineage 2, sublineage III, appeared in 2010, followed by the predominance of lineage 2, sublineage IV, in 2011 and later in Kolkata, India (15). Two isolates, IDH-00113 (referred to here as strain 13) and IDH-02387 (referred to here as strain 87), isolated in 2007 and 2009, respectively, represented lineage 1 and were predominant until 2009 (15). IDH-03329 (referred to here as strain 29), isolated in 2010, and IDH-03506 (referred to here as strain 06) and BCH-01536 (referred to here as strain 36), isolated in 2011, belong to sublineages III and IV, respectively, of lineage 2. Lineage 2 sublineage III strains were transient in Kolkata and observed only in 2010. Thereafter, sublineage IV strains of lineage 2 became predominant.

**FIG 2:**
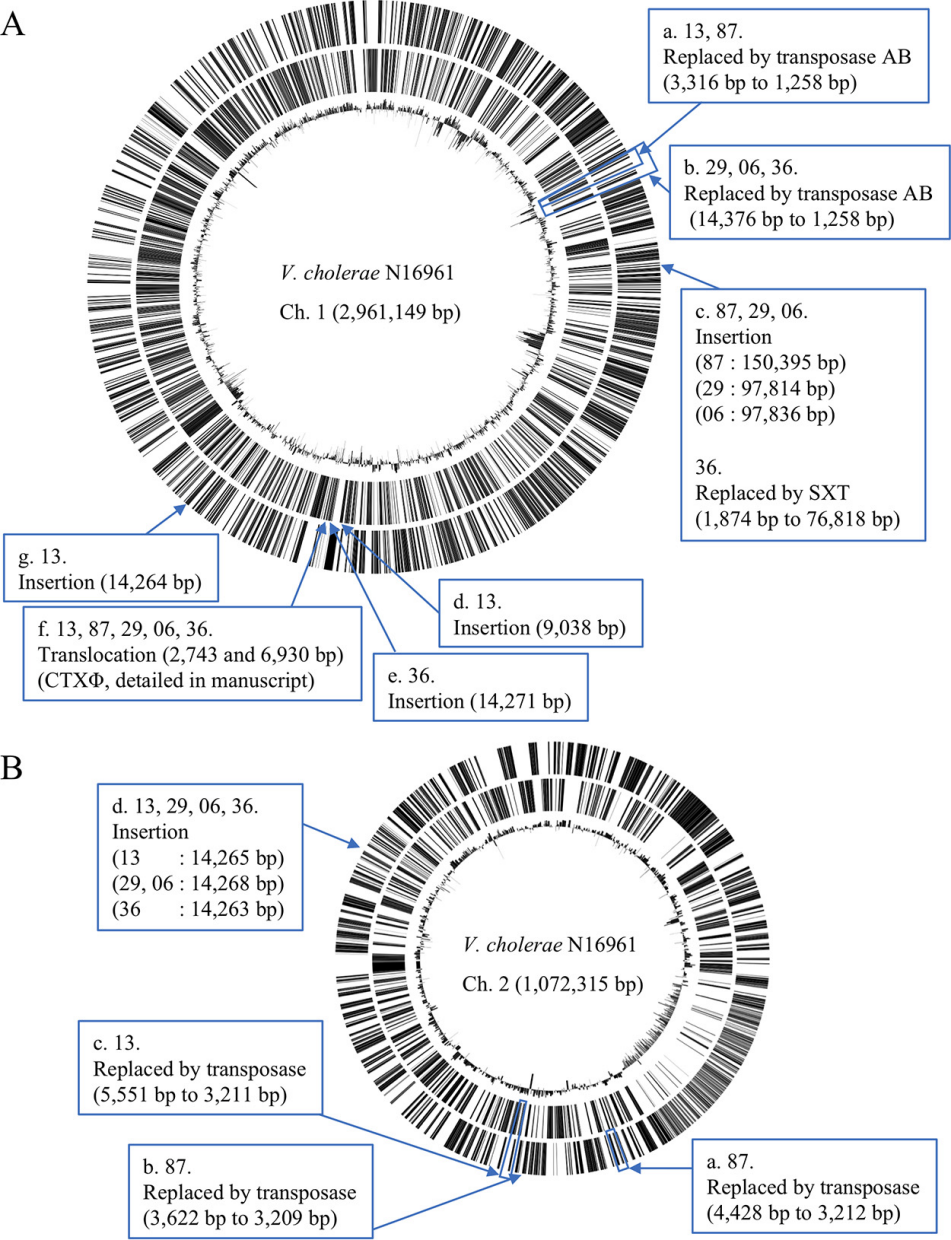
Structural variations in the chromosomes of V. cholerae. Replacements, insertions, and translocations of >2,000 bp compared with the N16961 genomic sequences, suggested on the basis of complete genomic sequences obtained in this study and confirmed by PCR, are illustrated. Circular chromosomal maps having genes on the plus strand, those with genes on the minus strand, and GC percent (from outside to inside) were generated using CiVi (Circular Visualization for Microbial Genomes [60]). A, larger chromosome; B, smaller chromosome.

Long-read genomic sequences of these strains were obtained using Oxford Nanopore MinION, and the nucleotide sequences were polished by short reads obtained by Illumina sequencing (15). Then, the complete genomic sequences were assembled and their chromosomal structures were compared with that of V. cholerae N16961, a prototype El Tor strain. The detected structural variations were verified through PCR amplification (data not shown). The confirmed differences of >2,000 bp are summarized in Fig. 2 and Table 1. The previously identified variations in the VSP-II genetic island, in which 3,343-bp and 14,376-bp regions were replaced by transposase genes in lineages 1 and 2, respectively, were confirmed in this study (15) (Fig. 2A, boxes a and b). Various integral and conjugative elements of approximately 150, 98, and 77 kbp were found in isolates 87, 29/06, and 36, respectively; these findings were consistent with those published in a recent report (22) (Fig. 2A, box c). Insertions of 9,038 bp, 14,271 bp, and 14,264 bp fragments including a gene encoding transposase in strains 13, 36, and 13, respectively, were identified (Fig. 2A, boxes d, e, and g). The DNA fragment was inserted in strain 36 into *zot*, a gene in the CTXΦ prophage genome, and consequently, the gene was divided (Fig. 2A, box e). The 14-kbp (approximately) sequences inserted in strains 13 and 36 were nearly identical to the origin-proximal regions, including VC0175 to VC0185, suggesting duplication and insertion of the fragment into the distal region (Fig. 2A, boxes e and g).

**TABLE 1:** Genomic variations in recent V. cholerae isolates over 2,000 bp in size

Position in Fig. 2	Position in N16961 genome		ORFs at position	Altered type	Altered strains	Size		Description of acquired sequence
	Start	End				Lost	Acquired	
Chr. 1^*a*^								
a	529,128	532,472	VC0495 to VC0500	Replacement	13, 87	3,343	1,258	VSP-II, transposase
b	529,128	543,505	VC0495 to VC0512	Replacement	29, 06, 36	14,376	1,257	VSP-II, transposase
c	703,929	703,944	VC0659	Insertion	87		150,395	SXT element
c	703,923	703,944	VC0659	Insertion	29		97,814	SXT element
c	703,929	703,944	VC0659	Insertion	06		97,836	SXT element
c	702,055	703,930	VC0658 to VC0659	Replacement	36	1,874	76,818	SXT element
d	1,545,050	1,545,057	VC1446	Insertion	13		9,038	Transposase
e	1,568,642	1,568,643	VC1548 (*zot*)	Insertion	36		14,271	Includes genes for transposase and phage integrase
f				Translocation	13, 87, 29, 06, 36			CTXΦ and RS1, detailed here
g	1,817,850	1,817,852	VC1683	Insertion	13		14,264	Includes genes for transposase and phage integrase

Chr. 2								
a	479,414	483,843	VCA0543 to VCA0545	Replacement	87	4,428	3,212	Transposase
b	556,942	560,565	VCA0623 to VCA0625	Replacement	87	3,622	3,209	Transposase
c	567,157	572,709	VCA0629 to VCA0638	Replacement	13	5,551	3,211	Transposase
d	634,743	634,744	VCA0695	Insertion	13		14,265	Includes genes for transposase and phage integrase
d	634,222	634,223	VCA0695	Insertion	29		14,268	Includes genes for transposase and phage integrase
d	634,743	634,744	VCA0695	Insertion	06		14,268	Includes genes for transposase and phage integrase
d	634,743	634,744	VCA0695	Insertion	36		14,263	Includes genes for transposase and phage integrase

a Chr., chromosome.

In addition to these insertions and replacements, translocation within the CTXΦ region in all recent isolates compared with the N16961 strain was detected (Fig. 2A, box f). The analysis of this structural difference is described in detail below. In the smaller chromosome, three replacements by a transposase-encoding gene (Fig. 2B, boxes a, b and c) and an insertion of approximately 14-Kbp fragment in strains 13, 29, 06, and 36 were detected (Fig. 2B, box d). This 14-kbp fragment was also almost identical to the region VC0175 to VC0185 in the chromosome 1 (Fig. 2A, boxes e and g), indicating duplication and insertion from larger to smaller chromosomes. In summary, the results indicated that the chromosomes of pathogenic V. cholerae strains were frequently replaced with mobile genetic elements and were highly diverse even in spatiotemporally close clinical isolates.

### Alteration of CTXΦ prophage arrays.

**FIG 3:**
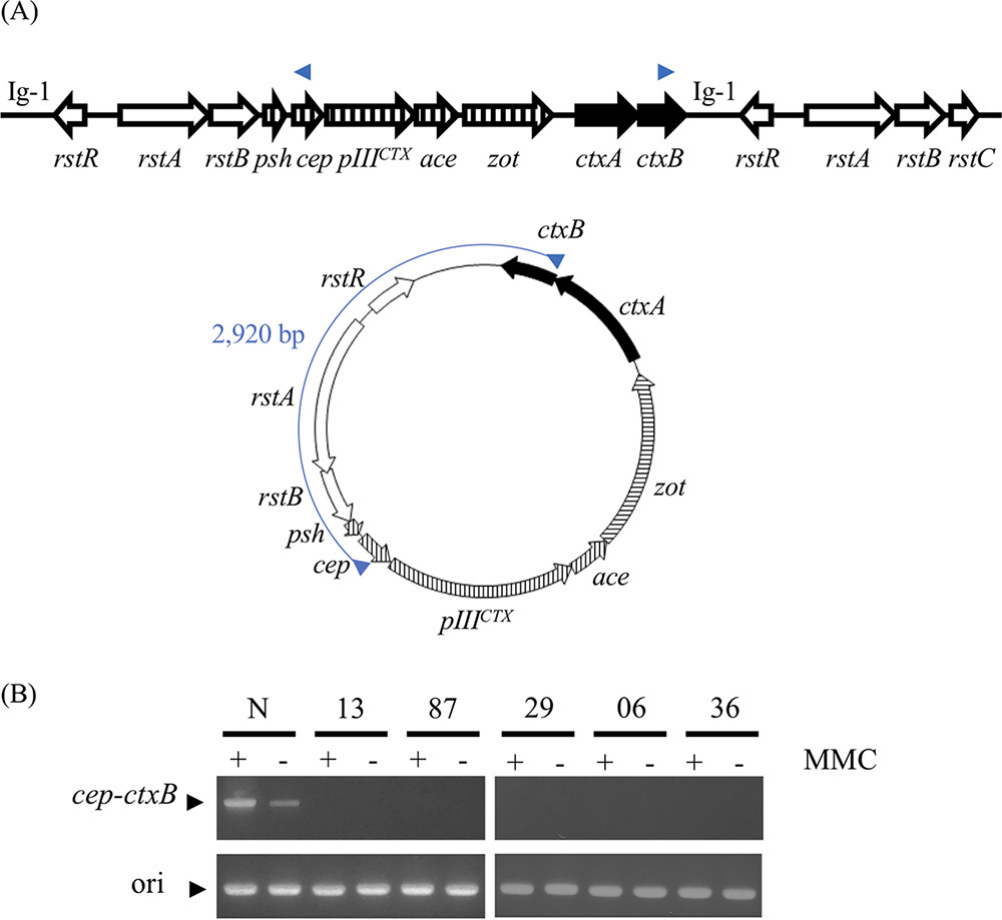
Detection of rolling-circle replication products of the CTXΦ prophage genome. Primers were designed in *cep* (P17) and *ctxB* (P16) in the CTXΦ prophage genome in inverse directions (A, top). DNA fragments of 2,920 bp were amplified only from circular rolling-circle replication products (A, bottom). (B) Amplified fragments from CTXΦ replication products and origin proximal genomic region as a control. MMC, mitomycin C induction; N, strain N16961.

We next focused on the alteration of the CTXΦ prophage array among the identified structural differences (Fig. 2; Table 1). The CTXΦ prophage genome in the El Tor biotype strains is usually found in tandem and/or followed by the related genetic element known as RS1, and this tandem array is essential for the replication and induction of CTXΦ (17) (Fig. 1A to D). In brief, the CTXΦ prophage genome was replicated by nicking two Ig-1 sites of CTXΦ and the following element by either tandem CTXΦ or RS1, i.e., Ig-1 upstream and downstream of the CTXΦ genome (Fig. 1A). The complete genomic sequences obtained in this study and after PCR confirmation indicated that all recent isolates possessed intact gene sets of CTXΦ prophage and RS1. However, RS1 was located at the upstream end of the CTXΦ genome, in contrast to the prototype El Tor strains (Fig. 1A and E). These arrays of the CTXΦ region possessed Ig-1, a nicking site for rolling-circle replication, only at the upstream end of the prophage genome. This observation suggests that the recent V. cholerae epidemic strains have lost the ability to replicate the CTXΦ genome and have led to the subsequent production of infectious phage particles. To confirm this possibility, a circular replication product of CTXΦ phage was specifically detected using PCR (Fig. 3A). The prototype El Tor type strain N16961 produced the replication product with and without mitomycin C induction (Fig. 3B). However, no such replication was detected in all recent isolates, even in the mitomycin C-induced condition (Fig. 3B). These results confirmed that recent V. cholerae strains are incapable of replicating the CTXΦ prophage genome.

### Factors required for replication.

**FIG 4:**
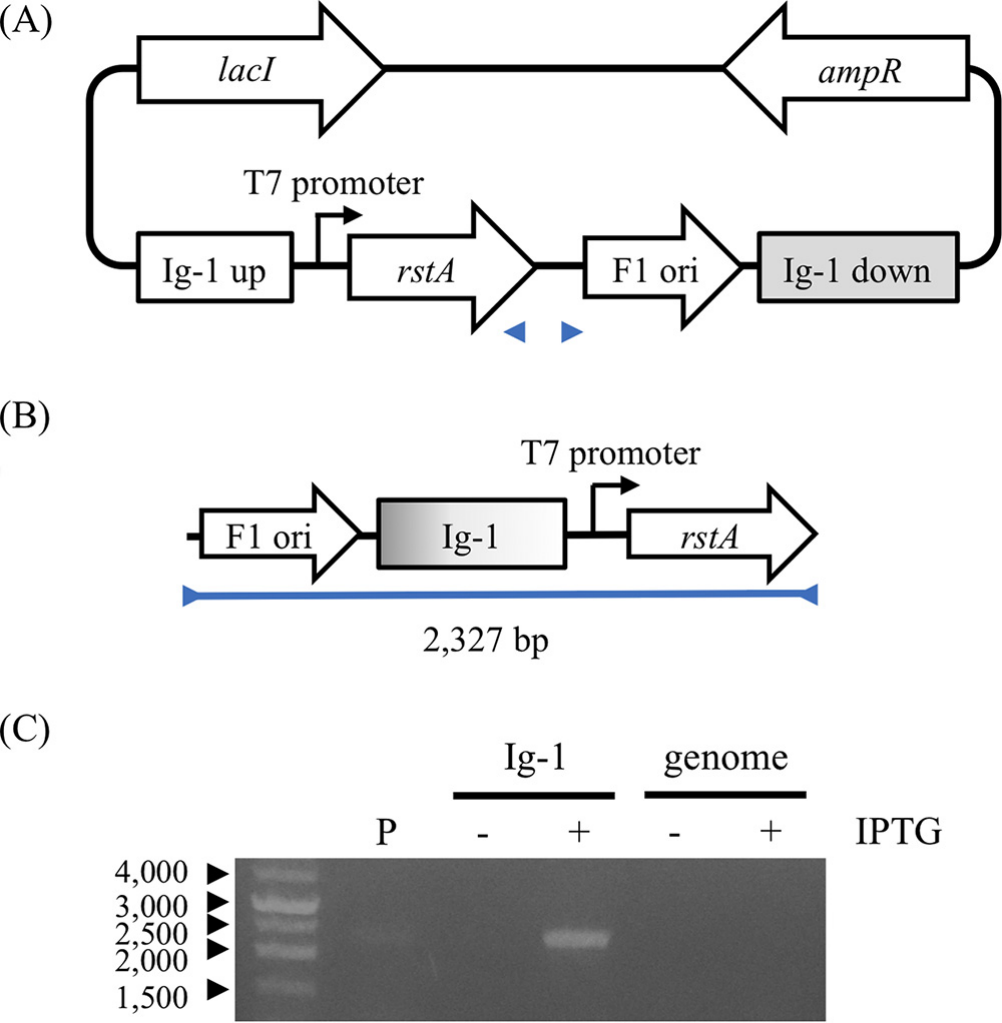
Reconstitution of rolling-circle replication by RstA in E. coli. *rstA* was cloned into pET-21a under the control of the T7 promoter. The Ig-1 regions of CTXΦ and RS1 were inserted upstream and downstream of *rstA*, respectively (A). The circular rolling-circle replication product was detected using primers in inverse directions (B and C). P, empty plasmid; genome, genomic sequences of V. cholerae instead of Ig-1 were cloned into the plasmid; IPTG, isopropyl-β-d-1-thiogalactopyranoside induction of *rstA* expression.

**FIG 5:**
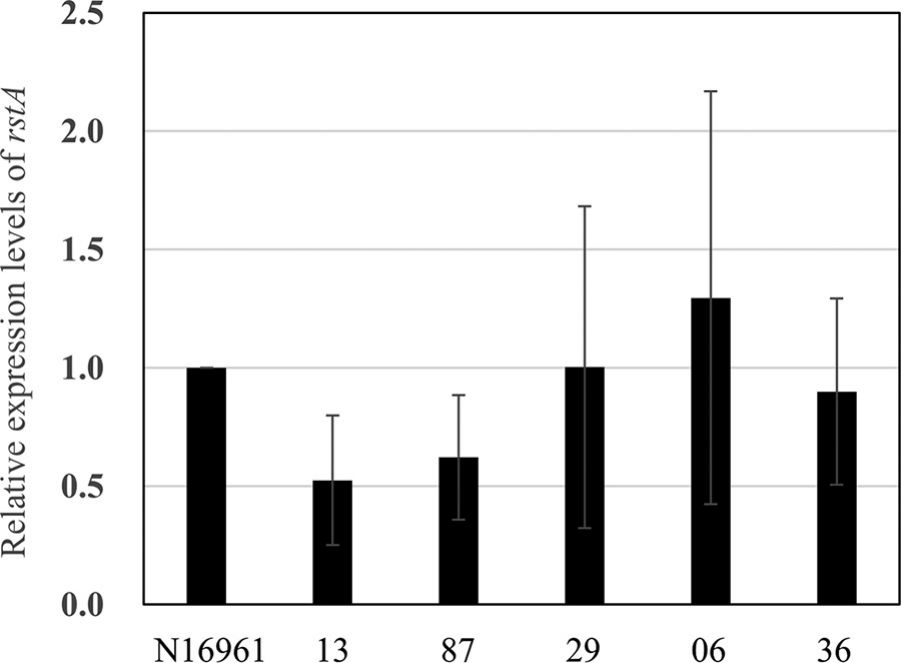
Expression levels of *rstA*. mRNA of *rstA* was quantified by reverse transcription and qPCR. Expression levels relative to that of N16961 are shown. Data points are averages from three independent experiments. Error bars represent standard errors.

It was reported that *rstA* is the only CTXΦ gene required for its replication in V. cholerae (19). We next investigated whether *rstA* is sufficient for the replication in the absence of CTXΦ and specific elements of V. cholerae. *rstA* was cloned into the plasmid pET-21a, and Ig-1 sequences were inserted upstream and downstream of the gene (Fig. 4A). *rstA* expression was induced in E. coli BL21 using IPTG (isopropyl-β-d-thiogalactopyranoside), and circular rolling-circle replication products were detected using PCR with the primers in the inverse direction (Fig. 4A and B). The V. cholerae genomic sequence was inserted instead of Ig-1 as a negative control. The replication was detected in Escherichia coli only in the presence of both *rstA* expression and Ig-1 (Fig. 4C). These results indicated that the two Ig-1 nicking sites as well as *rstA* expression were necessary and sufficient in V. cholerae and CTXΦ-specific elements for replication. Because all strains used in this study possessed Ig-1, *rstA*, and its upstream sequence identical to those of V. cholerae N16961, *rstA* expression levels in these strains were compared. *rstA* was mainly expressed in the exponential growth phase, and the expression decreased in the stationary phase in all tested V. cholerae strains (data not shown). Although the relative expression levels of recent isolates in the growth phase compared with N16961 showed some variation (0.5- to 1.3-fold), comparable *rstA* expression levels in all strains were confirmed (Fig. 5). These results indicated that all strains used in the present study possessed the necessary genetic elements for CTXΦ replication, and thus, the altered prophage array structure was supposedly responsible for the inability to replicate.

### Impact of CTXΦ replication.

**FIG 6:**
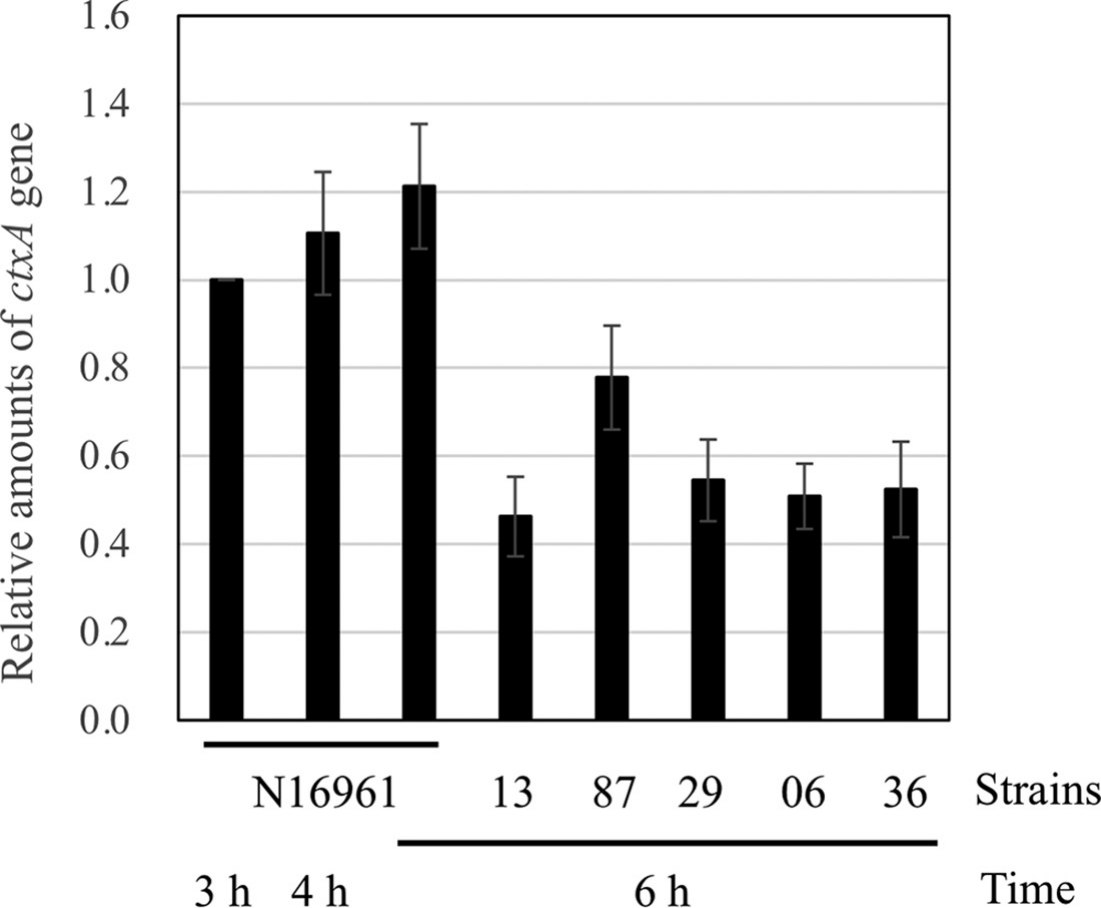
Quantification of *ctxA*. DNA fragment of *ctxA* was quantified, and the relative amounts are shown. “Time” indicates the number of hours of incubation. Data points are averages from five independent experiments. Error bars represent standard errors.

We estimated the number of CTXΦ phage produced from a single V. cholerae bacterium. Because CTXΦ is not a plaque-forming phage and phage particles are not detected as PFU, its genomic DNA was quantified instead of phage particles. All strains used in this study possessed a single copy of *ctxA* in the genome (Fig. 1A and E). Therefore, the DNA fragment of *ctxA* from an equal amount of extracted DNA was compared using quantitative PCR (qPCR) for the CTXΦ replication of positive and negative strains (Fig. 6). In the CTXΦ replication-positive N16961 strain, the amount of *ctxA* fragment increased slightly during stationary phase compared to that in the exponential growth phase. All the negative isolates from CTXΦ replication exhibited approximately half of the amount of the *ctxA* fragment in N16961 (Fig. 6). It is not clear whether a small cell population replicated the CTXΦ prophage genome many times or most cells replicated it only a few times. Nonetheless, these results indicated that prototype El Tor strain produces CTXΦ phage at number comparable to that of the cell population and that its impact on the dissemination of cholera cannot be ignored.

### Variation in CTXΦ arrays of known V. cholerae strains.

**FIG 7:**
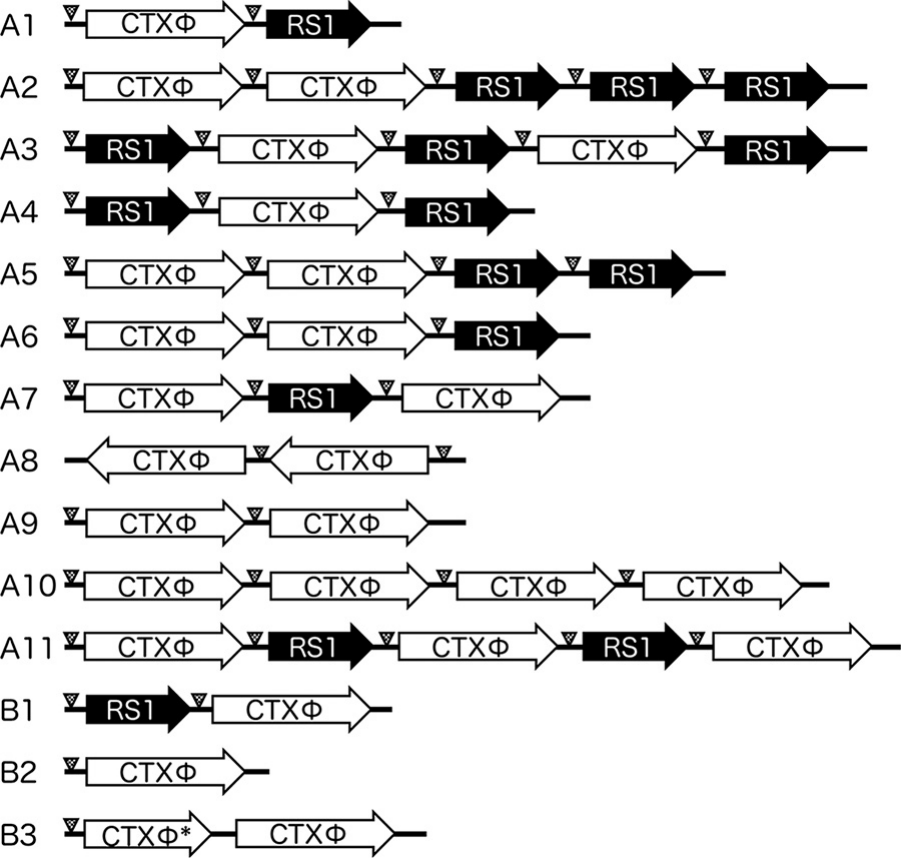
Variations in CTXΦ arrays in pathogenic V. cholerae isolates. The CTXΦ arrays of pathogenic V. cholerae strains listed in Table 2 are shown. Arrows labeled “CTXΦ” and “RS1” indicate the CTXΦ genome in the *rstR*-to-*ctxB* direction and the RS1 element in the *rstR*-to-*rstC* direction, respectively (Fig. 1A). The right side of the displayed array is followed by the *rtxA* gene. *, truncated CTXΦ genome lacking sequence downstream from the internal region of *cep*, as shown in Fig. 1 (classical). Triangles, Ig-1 region (167 bp), which is required for rolling-circle replication (20). A and B indicate the CTXΦ arrays, which are expected to be capable and incapable of replication, respectively.

**FIG 8:**
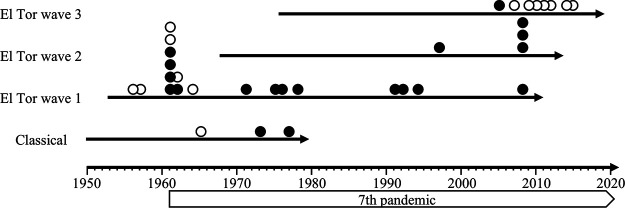
Schematic representation of CTXΦ productivity and years of isolation of V. cholerae. Years in which V. cholerae strains with replicative (black circle) and nonreplicative (white circle) CTXΦ arrays were isolated are indicated (Table 2). The biotypes and waves of each strain were determined on the basis of *ctxB*, *rstR*, and *rstA* genotypes (14).

The CTXΦ array patterns detected in 52 V. cholerae strains are depicted in Table 2 and Fig. 7. Data regarding the year of isolation and the complete genomic sequence or CTXΦ structures are available in the literature (23). Fourteen CTXΦ array patterns were identified from strains isolated between 1956 and 2015 from Asia, Africa, Latin America, and North America (Fig. 7; Table 2). Among the 14 CTXΦ array variations, 11 possessed Ig-1 at both the upstream and downstream ends of CTXΦ prophage genome (indicated with an “A” in Fig. 7), suggesting that these strains can produce infectious virions, whereas 3 CTXΦ arrays demonstrated defective replication when the structure was observed (indicated with a “B” in Fig. 7). Of note, non-CTXΦ-producing strains were isolated in 1965 and earlier, but, later on, CTXΦ-producing strains were reported to be more dominant worldwide for over 3 decades (Fig. 8; Table 2). The strain used in this study, i.e., those with the non-CTXΦ-producing CTXΦ structure type B1, appeared in 2007 in India, and this type of strain became predominant after 2009 in Asia, Africa, and Latin America (Fig. 8; Table 2). In summary, these data suggest that V. cholerae epidemic strains did not produce CTXΦ phage during the sixth and early seventh pandemics, but the pattern shifted to the CTXΦ-producing strains in the 1970s. This probably generated new pathogenic V. cholerae strains in the environment through CTXΦ phage infection during these periods, which lasted for over 3 decades. However, the pandemic strains were again found to have lost the ability to produce CTXΦ phage particles around 2010, indicating that the observed dissemination patterns are at a critical stage during the ongoing cholera pandemic.

**TABLE 2:** V. cholerae strains and CTXΦ sequences from the literature cited in the study

Strain	Yr	Location	CTXΦ structure	Biotype	Genome accession no.	Reference
A1M	1956	Bangkok, Thailand	B1	El Tor, wave 1		23
C5	1957	Makassar, Indonesia	B2	El Tor, wave 1	GCA_001887395.1	38
C7	1961	Sulawesi, Indonesia	A10	El Tor, wave 1		23
C1	1961	Sulawesi, Indonesia	A11	El Tor, wave 1		23
J6	1961	Sarawak, Malaysia	A7	El Tor, wave 1		23
J9	1961	Sarawak, Malaysia	A7	El Tor, wave 1		23
C2	1961	Sulawesi, Indonesia	A7	El Tor, wave 1		23
P2	1961	Philippines	A9	El Tor, wave 1		23
P3	1961	Philippines	A9	El Tor, wave 1		23
P4	1961	Philippines	A9	El Tor, wave 1		23
P16	1961	Philippines	A9	El Tor, wave 1		23
P18	1961	Philippines	A9	El Tor, wave 1		23
P31	1961	Philippines	A9	El Tor, wave 1		23
P7	1961	Philippines	B1	El Tor, wave 1		23
E9120	1961	Indonesia	B2	El Tor, wave 1	GCA_001887655.1	39
M25	1962	Moji, Japan	A4	El Tor, wave 1		23
CRC711	1962	Kolkata, India	B1	El Tor, wave 1	GCA_001887435.1	40
193	1962	Taiwan	B1	El Tor, wave 1		23
341	1962	Taiwan	B1	El Tor, wave 1		23
T10	1962	Taiwan	B1	El Tor, wave 1		23
T100	1962	Taiwan	B1	El Tor, wave 1		23
CRC1106	1964	Kolkata, India	B2	El Tor, wave 1	GCA_001887455.1	40
O395	1965	India	B3	Classical		17
A19	1971	Bangladesh	A1	El Tor, wave 1	GCA_001250235.2	14
E506	1973	Texas, USA	A8	Classical	GCA_001887475.1	41
N16961	1975	Bangladesh	A1	El Tor, wave 1	GCA_003063785.1	42
P27459	1976	Bangladesh	A4	El Tor, wave 1	GCA_013085125.1	42
M2140	1977	Australia	A9	Classical	GCA_001887635.1	43
E7946	1978	Bahrain	A3	El Tor, wave 1	GCA_013085165.1	42
C6706	1991	Peru	A1	El Tor, wave 1	GCA_009763945.1	44
C6709	1991	Peru	A1	El Tor, wave 1	GCA_013085105.1	45
A1552	1992	Traveler from Peru to California	A1	El Tor, wave 1	GCA_003097695.1	46
IEC224	1994	Belém, Brazil	A1	El Tor, wave 1	GCA_000250855.1	47
V060002	1997	Patient who traveled to Indonesia	A9	El Tor, wave 2	GCA_003574155.1	48
FJ147	2005	China, Fujian	A1	El Tor, wave 3	GCA_000963555.1	49
3528-08	2008	Texas, USA	A2	El Tor, wave 2	GCA_009762895.1	50
3566-08	2008	New Jersey, USA	A5	El Tor, wave 2	GCA_009763105.1	51
MS6	2008	Thailand-Myanmar	A6	El Tor, wave 1	GCA_000829215.1	52
3569-08	2008	Louisiana, USA	A8	El Tor, wave 2	GCA_009762985.1	53
2010EL-1786	2010	Artibonite, Haiti	B1	El Tor, wave 3	GCA_009665515.1	53
H1	2010	Haiti	B1	El Tor, wave 3	GCA_000275645.1	53
KW3	2010	Haiti	B1	El Tor, wave 3	GCA_001318185.1	54
TSY216	2010	Tak, Thailand	B1	El Tor, wave 3	GCA_001045415.1	55
DRC-193A	2011	Congo	B1	El Tor, wave 3	GCA_013085145.1	56
2012EL-2176	2012	Haiti	B1	El Tor, wave 3	GCA_000765415.1	57
HC1037	2014	Jacmel, Haiti	B1	El Tor, wave 3	GCA_002946655.1	58
CTMA_1441	2015	Mutwanga, Congo	B1	El Tor, wave 3	GCA_009799825.1	59

## DISCUSSION

V. cholerae is generally found in an aquatic environment, where it acquires unique characteristic features that makes it better adapted to a particular environment through the uptake of genetic molecules from natural resources, either through transformation or via interaction with other inhabitants (4). In this manner, the bacteria communicate with the toxigenic CTXΦ phage and integrate the genomic constituents of the phage irreversibly into their genomes so as to gain the toxic components of the phage genome, mainly *ctxA* and *ctxB*; therefore, the risk of this disease progression in humans has invariably increased (2).

The two naturally occurring V. cholerae biotypes possess CTXΦ phage-integrated genomes but with different arrangements and cellular functions (17). In recent years, novel variants of V. cholerae O1 have been found to emerge with altered *ctxB* genotypes and with higher pathogenic potency (5, 24). Past reports suggested that the classical V. cholerae strains could not produce virions, as they would not yield extrachromosomal CTXΦ DNA (17). Comparative genomics revealed that, despite having functional genes for the replication and production of phage particles, classical strains were unable to replicate the CTXΦ phage genome only because of the structural deficiencies in the CTXΦ prophage array (17). However, on the emergence of the seventh pandemic, the El Tor strains were found to possess functional CTXΦ phage genomes that could produce transducible virions (25). With time, different El Tor strains (atypical El Tor) were found to emerge from different places worldwide with modified genetic makeup of the CTXΦ prophage (5). These atypical El Tor strains were found to arise on the prototype El Tor genomic background only by replacing CTXΦ phages of different types (21). Thus, the wave 2 El Tor strains possessed tandem repeats of classical-CTXΦ-like prophages on their second chromosome (14). Other CTXΦ prophage-containing pathogenic variants of V. cholerae included V. cholerae O139, which harbored an extra copy of a different CTXΦ prophage located at the downstream end of the preexisting El Tor type CTXΦ prophage on the first chromosome (26). Faruque et al. demonstrated the presence of a different type of CTXΦ prophage array in the isolates of the Mozambique variant El Tor strains (27). These strains contained 2 copies of the classical CTXΦ prophages in the second chromosome, but they were unable to produce virions.

In this study, we demonstrated that recent V. cholerae clinical strains isolated in Kolkata were incapable of replicating the CTXΦ prophage genome and hence were not responsible for the production of infectious virions. Because CTXΦ is not a plaque-forming phage, genetic engineering of pathogenic V. cholerae to introduce an antibiotic-resistant gene into the CTXΦ genome is required to assay phage particle productivity (6). These analyses are expected to further confirm the conclusions of the present study. Nevertheless, the data in the present study strongly suggest that the recent V. cholerae epidemic strains do not produce infectious virions. In strain 36, a gene in the CTXΦ genome, *zot*, was disrupted by the insertion of a 14,271-bp fragment that included a transposase-encoding gene (Fig. 2A, box e). Zot is required for packaging and secretion of the phage as well as possessing enterotoxin activity (21). It may be suggested that the disruption of *zot* was allowed because phage secretion was no longer required in the strain incapable of replicating the CTXΦ genome.

The infection of V. cholerae O1 cells by CTXΦ requires toxin-coregulated pilus (TCP) as the receptor (6). Biogenesis of TCP is dependent on the *tcp* operon in *Vibrio* pathogenicity island 1 (VPI-1) on larger chromosomes. The first gene of the operon, *tcpA* encodes the major pilin subunit (28). Thus, the *tcp*-positive V. cholerae O1 strains are potential hosts for the CTXΦ infection to generate epidemic strains. We attempted to isolate V. cholerae O1 strains from lakes, ponds, and rivers in Kolkata, India, between 2014 and 2016 several times and characterized the strains. During this analysis, only one *ctxA*-positive strain was isolated, whereas 181 *tcpA*-positive strains were identified (data not shown). These observations suggest that many more potential host cells for the CTXΦ infection exist than the pathogenic V. cholerae O1 strains in environmental water. Therefore, the cholera pandemic caused by the CTXΦ-producing strains disseminated CTXΦ phage particles into the environment and possibly generated new pathogenic V. cholerae O1 strains. Thus, the results of the present study suggest that, during the seventh pandemic, the spread of cholera acquired a secondary disseminating route in which the secreted CTXΦ phage particles generated new pathogenic V. cholerae O1 strains in the environment (Fig. 8). It was reported that the El Tor type strains were less virulent than the classical type strains, but the recent variant exhibited increased production of toxins (29). It can be concluded that non-CTXΦ-producing strains with higher virulence were disseminated by the fecal-oral route by causing severe diarrhea and that CTXΦ-producing strains with lower virulence were spread both by the fecal-oral route and by the generation of new pathogenic strains in the environment because of CTXΦ infection. Thus, the prototype El Tor strains required the spread of CTXΦ productivity, while the classical type and the recent variant did not. On the other hand, CTXΦ productivity may be one of the reasons why the seventh pandemic continued for a longer period, i.e., for over half a century, than other pandemics. If this is the case, the appearance of the non-CTXΦ-producing El Tor strain brought a significant change that can be of great help to restrict the dissemination of V. cholerae and cholera worldwide. Epidemiological studies shall further confirm and reveal the effects of the change in CTXΦ productivity of V. cholerae found in the present study.

In addition, V. cholerae El Tor strains, which were more stable in the environment but less pathogenic than classical strains, acquired virulence properties of the hypervirulent classical strains in recent years. The first report of V. cholerae El Tor strains mentioned the acquisition of classical *ctxB* in the El Tor genomic background in the strains of Kolkata during the 1990s (10). Thereafter, the appearance of a new variant hyperpathogenic *ctxB* genotype (*ctxB7*) was first observed in the isolates of Kolkata during 2006, which attracted the attention of scientists after the Haitian cholera outbreak of 2010 (13). It was recently discovered that one of the major phenotypic characteristics of the classical biotype strain, i.e., polymyxin B sensitivity, was also transmitted to the El Tor biotype strains circulating in Kolkata (30, 31). A similar finding was made in this study with regard to the characteristic of the classical biotype strain, i.e., the inability to produce CTXΦ virions like the classical strains, although it involved a different mechanism. Thus, the recent trend of gaining classical biotype traits by El Tor biotype strains indicates that the new variant V. cholerae El Tor strains with hyperpathogenic characteristics adapt slowly to the environment and also evolve slowly; such strains can prove fatal to human beings and may lead to a more severe cholera outbreak situation in the near future worldwide.

## MATERIALS AND METHODS

### Strains used in the study.

The strains were isolated from patients with cholera in Kolkata, India, between 2007 and 2011 and were phylogenetically analyzed as described previously (15). Strains IDH-00113 (referred to here as strain 13) and IDH-02387 (strain 87) isolated in 2007 and 2009, respectively, belong to lineage 1, which was predominant in Kolkata until 2009. Strain IDH-03329 (strain 29), isolated in 2010, as well as IDH-03506 (strain 06) and BCH-01536 (strain 36), isolated in 2011, were classified into lineage 2, sublineages III and IV, respectively. It was revealed that lineages 1 and 2 were concurrently prevalent between 2007 and 2009, while lineage 2-III appeared in 2010, followed by the predominance of lineage 2-IV in 2011 and later (15).

### Plasmid construction.

The oligonucleotide primers used in the study are listed in Table 3. To construct the *rstA* expression plasmid with Ig-1 or control genomic sequence, the primer pairs P1/P2, P3/P4, P5/P6, and P7/P8 were used to amplify the Ig-1 of CTXΦ (Ig-1 up), Ig-1 of RS1 (Ig-1 down), an N16961 genomic region of approximately 1.5 Mbp (1.5 genome), and a 1.1-Mbp (1.1 genome) region of the larger chromosome, respectively. Inverse PCR was performed using the primer pair P9/P10 and pET-21a as a template, followed by ligation using a seamless ligation-independent cell lysate (32) with the Ig-1 up or 1.5 genome fragment. Inverse PCR was performed again using the resulting plasmids as a template, with the primer pair P11/P12 ligated with the Ig-1 down or 1.1 genomic fragment. The resulting plasmids were digested using NdeI/XhoI and ligated with an NdeI/XhoI-digested *rstA* fragment, which was amplified with primer pair P13/P14. Constructed plasmids were verified via sequencing. The plasmids were propagated in Escherichia coli DH5α, and *rstA* expression was induced in E. coli BL21.

**TABLE 3:** Primers used in this study

Name	Sequence (5′→3′)
P1	TGCGTCCGGCGTAGACTAAACCTAGAGACAAAATG
P2	TCGAGATCTCGATCCAGCATCTTAAATCATGGTGC
P3	ACTTTTCGGGGAAATCAAACATGTATTACTGCAAG
P4	AGGGGTTCCGCGCACAGCATCTTAAATCATGGTGC
P5	TGCGTCCGGCGTAGATCTTGTAATTGAATTATCCG
P6	TCGAGATCTCGATCCTCTAAGTCCAACTTCCTCGC
P7	ACTTTTCGGGGAAATAATGGACGTATTCTGTCACC
P8	AGGGGTTCCGCGCACGACGTCAGGTCAGGTTGATC
P9	GGATCGAGATCTCGATCCCG
P10	TCTACGCCGGACGCATCGTG
P11	ATTTCCCCGAAAAGTGCCAC
P12	GTGCGCGGAACCCCTATTTG
P13	GGAATTCCATATGAAAAAGCAGATTTTCAC
P15	CCGCTCGAGATCACCCATAATTTCATCAATTAAC
P16	TGAAAGGATGAAGGATACCC
P17	ACCGTATCTTTACTGGTGCC
P18	CTCCAAGCGTTCCATCATG
P19	GCGCATAAGTCCGATTTGTC
P20	GGTACTGAAGGGTCTGGATG
P21	CGATGTCTTTACAGTAACCTGC
P22	CAGATTCTAGACCTCCTGATG
P23	TACACCTAGACTTTGGGTTT

### Genome sequencing.

The genomic DNA of V. cholerae strains was extracted using the DNeasy blood and tissue kit (Qiagen) as per the manufacturer’s instructions. Nanopore-based DNA sequencing was performed using the Native Barcoding Expansion 1-12 (EXP-NBD104; Oxford Nanopore Technologies [ONT, Oxford, UK]) and a ligation sequencing kit (SQK-LSK109; ONT), and DNA was loaded onto the MinION sequencing apparatus flow cell (R9.4.1; FLO-MIN106D; ONT) as per the manufacturer’s instructions. The raw reads were base called (i.e., electronic signals were converted to the corresponding base sequence of the DNA strand) using Albacore software (ONT). The DNA sequence reads obtained were separated on the basis of the barcode sequence of each strain, and the adapters were trimmed off by using the Porechop software (33). Circular chromosomal sequences were assembled from the obtained reads with at least 30-fold coverage of the V. cholerae genome using flye (34) or unicycler (35), and the sequences were then polished by short reads (15) using Pilon software (36).

### Detection of structural variation in genomes.

The obtained genomic sequences were compared with those of V. cholerae N16961 by 500 kbp each using Easyfig software (https://mjsull.github.io/Easyfig/). The structural variants were further compared with the help of the dot plot using BLAST. These variants were verified using PCR by amplifying the upstream and downstream ends of the altered region (data not shown).

### Rolling-circle replication.

V. cholerae strains were incubated in alkaline peptone water at 37°C. To this, 20 ng/ml of mitomycin C was added to induce CTXΦ prophage (37) at an optical density at 600 nm (OD_600_) of 0.2. After 7 h of initiation of incubation, total DNA was extracted using the phenol-chloroform method. The total DNA was adjusted to 100 ng/μl and used as a template for PCR with the primer pair P16/P17.

### Relative expression levels of *rstA*.

Total RNA was extracted using the Quick-RNA MiniPrep Plus kit (Zymo Research) as per the manufacturer’s instructions. Sixteen nanograms of extracted RNA was subjected to reverse transcription with random primers using iScript reverse transcription supermix for performing reverse transcription-quantitative real-time PCR (RT-qPCR) analysis (Bio-Rad). cDNA for *rstA* and the gene VC0015 (encoding gyrase) were quantified by qPCR (PowerTrack SYBR green master mix; Thermo Fisher) with the primer pairs P18/P19 and P20/P21, respectively. VC0015 was used to normalize the expression level of *rstA*.

### Quantification of *ctxA* DNA.

The V. cholerae strains were incubated in alkaline peptone water at 37°C for the indicated time periods from 3 to 6 h. DNA was extracted and adjusted to 0.6 ng/μl and then subjected to qPCR with the primer pair P22/P23. The relative quantity of *ctxA* DNA with respect to the N16961 strain at 3 h was determined.

### Data availability.

Nucleotide sequence data for larger and smaller chromosomes of strains IDH-00113, IDH-02387, IDH-03329, IDH-03506, and BCH-01536 generated in this study are available in the DDBJ database under accession numbers AP024549/AP024550, AP024551/AP024552, AP024553/AP024554, AP024555/AP024556, and AP024547/AP024548, respectively.
